# ProTaxoVis—protein taxonomic visualisation of presence

**DOI:** 10.1186/s12859-025-06146-9

**Published:** 2025-05-19

**Authors:** Yin-Chen Hsieh, Mathias Bockwoldt, Ines Heiland

**Affiliations:** Department of Arctic and Marine Biology, Faculty of Biosciences, Fisheries and Economics, UiT Arctic University of Norway, 9037 Tromsø, Norway

**Keywords:** Protein distribution, Protein taxonomy, Enzyme presence-absence, Sequence query, Comparative pathway analysis

## Abstract

**Background:**

Protein presence information is an essential component of biological pathway identification. Presence of certain enzymes in an organism points towards the metabolic pathways that occur within it, whereas the absence of these enzymes indicates either the existence of alternative pathways or a lack of these pathways altogether. The same inference applies to regulatory pathways such as gene regulation and signal transduction. Protein presence information therefore forms the basis for biological pathway studies, and patterns in presence-absence across multiple organisms allow for comparative pathway analyses.

**Results:**

Here we present ProTaxoVis, a novel bioinformatic tool that extracts protein presence information from database queries and maps it to a taxonomic tree or heatmap. ProTaxoVis generates a large-scale overview of presence patterns in taxonomic clades of interest. This overview reveals protein distribution patterns, and this can be used to deduce pathway evolution or to probe other biological questions. ProTaxoVis combines and filters sequence query results to extract information on the distribution of proteins and translates this information into two types of visual outputs: taxonomic trees and heatmaps. The trees supplement their topology with scaled pie-chart representations per node of the presence of target proteins and combinations of these proteins, such that patterns in taxonomic groups can easily be identified. The heatmap visualisation shows presence and conservation of these proteins for a user-determined set of species, allowing for a more detailed view over a larger group of proteins as compared to the trees. ProTaxoVis also allows for visual quality checks of hits based on a coverage plot and a length histogram, which can be used to determine e-value and minimum protein length cutoffs. Tabular output of resulting data from the query, combined, and heatmap building step are saved and easily accessible for further analyses.

**Conclusions:**

We evaluate our tool with the phosphoribosyltransferases, a transferase enzyme family with notable distribution patterns amongst organisms of varying complexities and across Eukaryota, Bacteria, and Archaea. ProTaxoVis is open-source and available at: https://github.com/MolecularBioinformatics/ProTaxoVis.

## Background

Sequence variation at the genetic level drives molecular evolution, but sequences originating from common ancestral sources tend to preserve a degree of similarity. This is because sequence conservation is necessary to preserve function, as is observed in homologous protein sequences. Since proteins are responsible for biological function, groups of proteins that operate together also tend to evolve and be inherited together [1, 2]. These patterns form the backbone of sequence-based phylogenetics, which aims to trace evolutionary topologies of sequences across organisms. Many bioinformatic tools [3, 4] apply this to construct tree-based overviews of the course of evolution that has brought about the distribution of proteins and functions in species observed today.

Constructing phylogenies from protein sequences has as prerequisite the presence of protein-encoding genes in the genome of a target species, and utilises sequence conservation as an indicator of evolutionary relationships between these species. Gene presence can be directly linked to the presence of proteins or other genetic products, therefore gene presence largely implies protein presence. In general, a map of protein presence across the proteome of a species of interest provides strong evidence as to which molecular functions the species carries out [1, 5–7] or serves as an evolutionary trace of functions the species used to carry out. This would arise when gene duplication events lead to loss-of-function in protein products, for example. Put all together, it follows then that protein presence information compared across species gives rise to evolutionary patterns of conservation and divergence that are used to further add to a sequence-based phylogenetic history of a taxonomic group.

Construction of large scale phylogenies from protein presence-absence is increasingly made possible by the volume of genomic data in modern databases, as well as the availability of accurate sequence querying tools. Since the characterisation of the first genome in the late 1970 s, the mass of available genomic data has exponentially grown, spanning over all kingdoms with more than 100,000 genera to encompass an estimate of around 500,000 species (July 2022 GenBank Statistics [8, 9]). This amounts to upwards of 250 million nucleotide sequences stored in databases such as RefSeq [10] and GenBank [9]. Coupled with the growth of high-throughput data generated by proteomics, metabolomics, and other -omics studies, genomic databases enable extraction of global perspectives on evolutionary patterns, and the computational work behind this is done primarily via bioinformatic sequence querying tools such as FASTA [11], BLAST [12], HMMER [13], and MMseqs2 [14], to name just a few. Such tools comb through databases via sequence similarity searches to extract potentially homologous sequences, or hits. Hits with alignment scores above thresholds for significant similarity are taken as proxy for biological presence of the gene or protein in the hit’s organism. In this way, a single sequence query’s hits span over a set of organisms that harbour the same or a close relative of the gene or protein, and the comparison of this presence information between organisms can be used, as mentioned above, to derive evolutionary histories. Combining information from multiple queries for various target sequences broadens the overall picture of evolutionary history.

The utility of presence-absence information is demonstrated by phylogenetic profiling, where binary presence-absence signatures of proteins are constructed from sequence database queries and compared across genomes of varying evolutionary divergence in order to determine the function of newly sequenced proteins or to map out potential protein-protein interactions. Pellegrini et al. [1] were the first to show that using *E. coli* gene presence-absence patterns in bacterial species was a viable method to assign functional annotation to newly discovered proteins. This was possible based off of the assumption that proteins that function together co-evolve and are co-inherited, leaving a trace in their presence-absence profiles. Much work has been done since to expand on this idea [15], and phylogenetic profiling is now used for an array of biological prediction purposes, from predicting human gene function [16, 17] to protein domain interaction [18] and molecular pathways [19].

In this work we present our tool, ProTaxoVis, Protein taxonomic visualisation of presence, that is inspired by traditional phylogenetic profiling approaches and focuses on showing a scalable taxonomic representation by combining information from multiple queries to generate an easily visualisable, global overview of presence patterns for a set of user-determined proteins, in both taxonomic trees and a heatmap formats. These visualisations show any taxa-specific preferences for certain proteins, as well as the conservation of proteins when compared to the query. This overview also allows for investigating taxonomic coverage in selected databases, and can be used to reveal scientific bias towards certain protein groups. This allows for a meaningful comparison of presence patterns for the purposes of signaling or metabolic pathway analysis, as the tool has demonstrated for the NAD [20, 21] and mTOR [22] pathways, but the potential applications are not limited to this scope, since protein distribution patterns are generalisable to investigate a multitude of biological questions.

## Implementation

### Architecture

ProTaxoVis is a command-line based software, consisting of three tools and one importable module. The first tool, *taxovis*, is a workflow for the main analysis of the software, and exists both as a command-line tool and a module. *taxovis* starts by performing sequence querying through calls to an external querying tool, and then retrieves, parses, and combines the resulting hits. The results are then used to build a taxonomic tree, an interactive heatmap, and also to plot coverage and sequence length plots for quality check of query results. ProTaxoVis’s second tool is a tree-specific pipeline, *taxotree*, which manages and displays trees built from *taxovis*. The third tool, *blast2fasta*, provides an additional functionality to collect query hit sequences into one file for alignment purposes. ProTaxoVis manages taxonomic information through its *TaxFinder* module, which is integrated into both *taxovis* and *blast2fasta*.

### Dependencies

ProTaxoVis is written in Python, and is compatible with Python 3.6+. Sequence querying is done with BLASTp [12], either through the command line tool BLAST+ [23], or through the NCBI web server. Query results are handled by the data management libraries biopython [24], numpy [25], scipy [26], and pandas [27]. ETE 3 Toolkit [4] is used for tree construction and manipulation. Plotting of sequence hit coverage and lengths is done with matplotlib [28]. Installation instructions, information on dependencies, and source code for the analysis are available at https://github.com/MolecularBioinformatics/ProTaxoVis.

ProTaxoVis uses the TaxFinder module to interact with NCBI taxonomic IDs from query results, therefore TaxFinder must also be installed in order for ProTaxoVis to run. TaxFinder contains functions to retrieve species names and information from taxonomic IDs, as well as functions to extract full taxonomic lineages from an ID. Through ProTaxoVis, users don’t use TaxFinder directly. However, the TaxFinder module can also be used as a standalone package for taxonomic ID information retrieval purposes. Installation instructions for TaxFinder can be found on the Github page (https://github.com/MolecularBioinformatics/taxfinder).

### General workflow

**Fig. 1:**
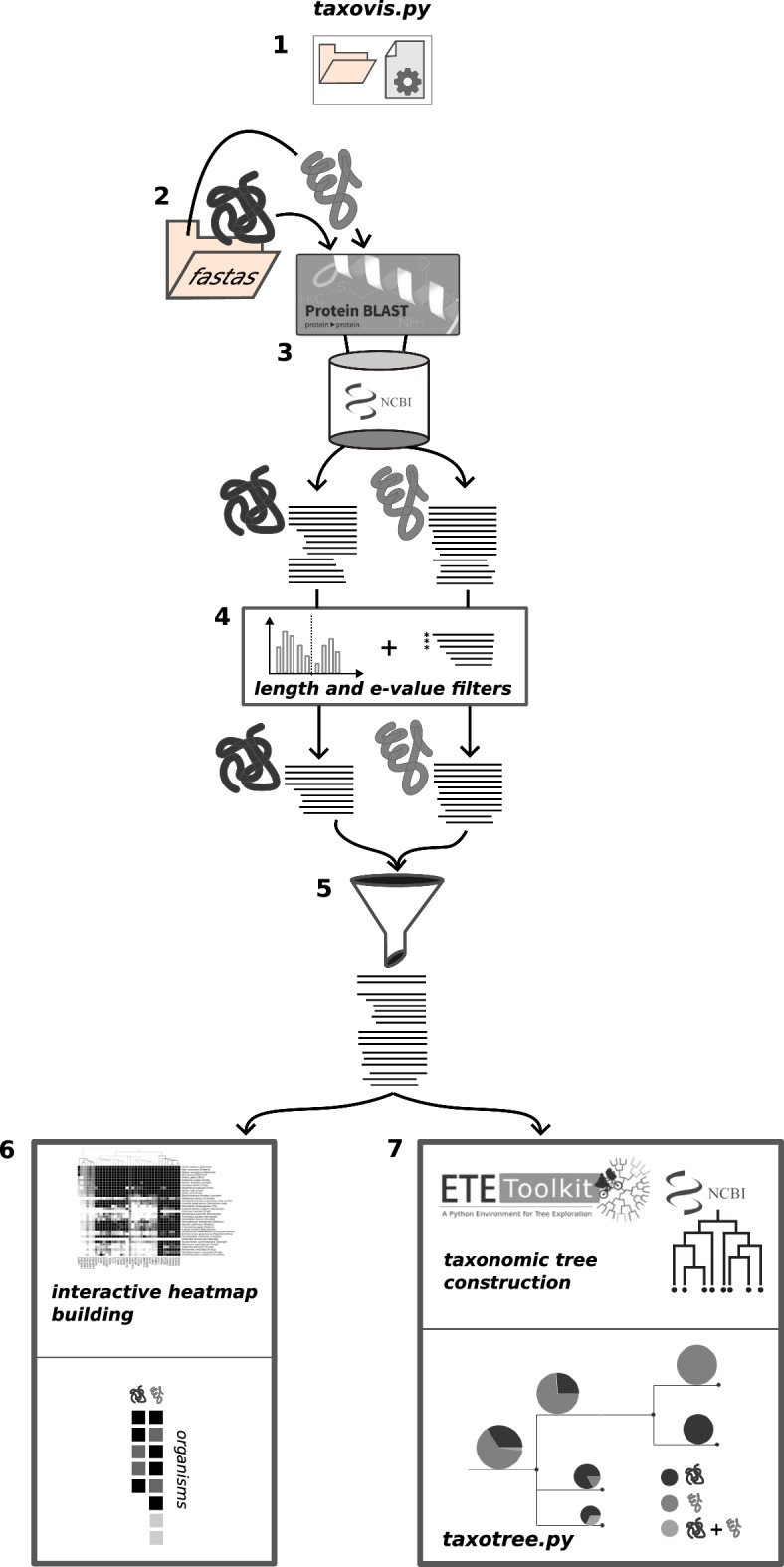
ProTaxoVis Workflow: First seven steps of a typical run with *taxovis* (1) initialisation of the folder structure to create necessary subfolders and configuration files, (2) retrieval and storage of seed protein sequences, (3) BLASTp query step of each individual protein, (4) setting length and e-value cutoffs and filtering each sequence set based on cutoffs, (5) combination of all remaining hit sequences, (6) generation of interactive heatmap, and (7) construction of taxonomic tree and visualisation with *taxotree*

The general workflow of ProTaxoVis is *taxovis* followed by *taxotree*. The workflow can be further divided as follows: initialisation, configuring input, choosing input seed sequences, sequence querying, query result processing, tree building, heatmap building, and visualisation. The steps are detailed in order below, and represented in Fig. 1.

ProTaxoVis requires a pre-defined folder structure containing several configuration text files, which is set up with *taxovis*’ initialisation function. This creates two folders, *blastresults* and *fastas*, which are used to store query results and seed sequences, respectively. The necessary configuration files are created, and have to be filled out manually by the user accordingly.

After preparation of the workspace with *taxovis*’ initialisation function, which creates the folder structure and configuration files necessary to begin the workflow, configuration files should be filled out. The configuration files store user defined input parameters that are required by ProTaxoVis at each step, and are described below:

- proteinlist.txt: two-column list of protein name and sequence file name
- limits.txt: e-value threshold and minimum length cutoffs for each protein (further discussed in query result processing section)
- heatmap_config.txt: sets the colors, hierarchical clustering linkage method for heatmap, and organisms to be shown
- tree_config.txt: sets the proteins (up to 3) to be visualised in the tree for the current run, and pie-chart colors
- tree_to_prune.txt: sets the taxa and partitions to show on the tree

The individual configuration files will be discussed further in their respective sections.

The choice of input sequences, or seed sequences, is entirely up to the user. Sequences should be protein sequences that are of interest because of either known or suspected involvement in a biological function, such as a metabolic pathway, for which the user is interested in obtaining a taxonomic overview. Chosen seeds should have decent annotation quality, ideally as confirmed sequences in a manually-curated database such as UniProt’s Swiss-Prot [29], to ensure correctness of sequence annotation. Correctness of seed sequence annotations ensures that query results represent the proteins of interest, which is often difficult in cases where seeds are highly similar to each other and could be mislabelled, as is often seen in the case of paralogous proteins. Paralogs require special consideration when used as a query and are dealt with at the filtering steps of the workflow. Seed sequences also should, dependent on the scientific question, be taken from a wider variety of taxonomic groups. This means that each protein has a set of seeds consisting of organisms that span over a sufficient evolutionary distance, having a sufficient level of divergence from the common ancestor.

In practice, this results in a set of model organisms being selected as seeds for each protein, provided that the protein is indeed expressed by these organisms, since they provide the most suitable sequence characteristics in terms of quality and correctness. Seed sequence extraction is done by the user, externally from ProTaxoVis, and sequences should be stored in FASTA format in the fastas folder prior to proceding with the workflow. Sequence file names and their corresponding proteins should be logged in the proteinlist.txt configuration file.

At the sequence querying step, the goal is to gather as many hits as possible such that the largest query space is sampled, and allow later filtering steps to reduce the set of hits through user-determined cutoff thresholds. BLASTp is used as the sequence query tool, and searches are conducted against any protein database that is indexed for BLASTp, or most commonly the NCBI nonredundant (nr) protein database (29), as it encompasses the main protein sequence databases from GenBank [9], RefSeq [10], and UniProt [29]. One could also query against each of these individual databases separately. The choice of database to search against ultimately depends on the desired level of completeness and coverage at the genomic level. So, if the query needs to be against high quality, whole genome sets, RefSeq and UniProt SwissProt would be recommended. Although BLASTp is an external step, ProTaxoVis provides a wrapper for the service to send the query sequence remotely to the NCBI server. This is done via biopython’s *NcbiblastpCommandline* functionality. Alternatively, the user can submit queries directly to the webserver, or locally with BLAST+. BLASTp parameters are mainly kept to the defaults, which are: BLOSUM62 alignment scoring matrix, word size of 6, gap-open cost of 11, and a gap-extension cost of 1. The e-value threshold can be set at 0.001, and the max target sequences should ideally be set to the max of 5000 (direct run on web server); 20,000 if run through ProTaxoVis’s wrapper or locally in BLAST+. The choice of e-value threshold does not need to be too stringent at this step. If one were to request 20,000 max target sequences, it is also better to raise the e-value to 0.01 or even 1, to allow more sequences to be returned. Query results should be written in XML2 format, and stored into the blastresults folder of the working directory. ProTaxoVis further parses these files into tables of tab separated value (TSV) format, stored in the resulttables folder.

**Fig. 2:**
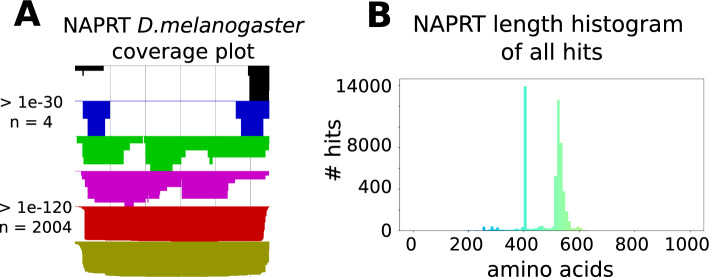
NAPRT Coverage Plot and Length Histogram: **A** The hit coverage plot of *Drosophila melanogaster* shows all hits sorted by significance, e-value, and aligned back to the original sequence, as is similar to the “Graphic Summary” view of BLAST output on the web server. A more stringent cutoff could be set at 1e-120 to include only sequence hits that are more complete, as seen through the alignment to the query. **B** Histogram of lengths of all sequence hits to all NAPRT seeds. Here two peaks are shown, one at 400aa and the other just before 600aa. The cutoff of 200 includes both peaks. If there is prior knowledge on the lengths of the desired hits, one could opt for a stricter length cutoff

Once BLAST query results have been collected for each protein, a quality check of these hits is necessary to determine the filter parameters to reduce potential inaccuracies or false positives in these hits. Filter parameters are set by the user upon review of quality check plots. Two types of plots are generated for this purpose - a histogram showing the sequence length distribution within a set of hits, and a coverage plot of hits that shows how much of each hit was aligned to the query, sorted according to number of hits falling into six e-value intervals of increasing significance. Using these two types of plots as a visual guide, the user can decide on the length and e-value cutoffs to impose on the query results such that unwanted hits can be sorted out. A length cutoff allows for filtering out hits that are too short compared to the query protein, and an e-value cutoff given the coverage sets the appropriate significance level at which to accept a hit with the assurance that important domains are still included. Examples of these plots are shown in Fig. 2, with nicotinate phosphoribosyltransferase (NAPRT) as an example query. Here a reasonable but more generous length cutoff could be set at approximately 50% of the seed protein sequence length, 200 aa, and a more stringent e-value could be 1e-120, to only include full sequence hits. ProTaxoVis’s default length and e-value settings are 50 amino acids and 1e-30, respectively. The default e-value is, in practice, good enough to exclude most low-quality aligned sequence hits, but the length cutoff should be adjusted per protein, based on around a minimum of 50% sequence length of the query or higher. The stringency of the length threshold is best set also with regards to how many hits have been returned. Length and e-value cutoffs are set in limits.txt.

An additional consideration when setting the e-value cutoff is the occurrence of cross-hits between two queries for different proteins. This is when hits corresponding to one protein appear in the set of hits of the other, and tends to occur when two proteins are highly similar (e.g. paralogs). Since the inclusion of hits to another query is not desired and will confound subsequent visualisation results, the e-value cutoff should be set for both proteins at the more stringent e-value in which the first cross-hit was found, such that any hits of less significance for both sets of hits will be excluded. ProTaxoVis performs the cross-check and displays e-values of any cross hits in the form of a similarity matrix in matrix.csv. A value of 0 indicates no cross-hit was found. Therefore, the ideal e-value cutoff should be set based on the lowest value (highest significance) between comparison of the coverage plot and the cross-hit matrix.

Once the length and e-value cutoffs have been set, ProTaxoVis filters each set of hits. Then, at the combination step, filtered sets corresponding to the same protein (from queries of different seeds) are combined into tabular form and stored in the folder named: combinedtables. This combination step also creates non-redundant lists of organisms (in names text files) and their taxids, per protein and also overall, combining results from all proteins. In such a way, the query result overview is consolidated and stored at this step.

ProTaxoVis builds an interactive heatmap to visualise presence-absence of proteins for a user-determined set of organisms. To generate the heatmap, the chosen set of organisms is clustered per protein according to the -log(e-value) of each hit sequence, which is an integer representing the amplitude of the significance of the hit, set with an upper bound at 200. Significance of the hit is indicated in the heatmap in grayscale, where a lighter gray is less significant and the darkening gradient represents increasing significance. Heatmaps with only black or white have the e-value of the best hit shown. Clustering is done via Scipy’s hierarchical clustering function, scipy.cluster.hierarchy [26], with the default linkage method set to centroid. Other linkage options, in addition to coloring of the heatmap, can be set by the user in the respective configuration file, heatmap_config.txt. The heatmap is written in HTML and JavaScript, and is thus viewable in any standard web browser.

ProTaxoVis’s interactive heatmap shows the grouping of organisms based on the presence-absence patterns of the protein set (vertical axis), with the proteins grouped based on the cluster dendrogram such that proteins with similar presence-absence patterns appear consecutively (horizontal axis). The user can toggle the heatmap display to show proteins listed alphabetically instead of in clustered format, and also to show binary black-white presence-absence (absolute) instead of grayscale (relative). The heatmap also allows for slider adjustment of displayed presence-absence based on e-value levels, and this is only available when the view format is toggled to absolute. The choice of absolute or relative visualisation depends on the question at hand and whether a single protein view is desired, or a comparison of multiple different proteins. For seed sequences from the same species of different proteins: If a user wants a broad overview of which species returned hits to the given set of seed sequences the absolute view is intended. Then, if the user requires a view into more closely related organisms from a certain seed protein, using the relative value could show how much the hits have diverged from each other. For seed sequences from different species of the same protein or multiple proteins: Care should be taken when interpreting results as the seed protein that a hit corresponded to should be verified. This can still provide a broad, non-specific overview of protein presence in a certain group. Either way, ProTaxoVis’s interactive heatmap is designed for visualising presence-absence patterns over a large set of proteins and a large user-determined set of organisms, and is meant to serve as a complement to the taxonomic tree.

ProTaxoVis generates its presence taxonomic tree with the Python-based ETE Toolkit [4] and NCBI Taxonomy [8]. The topology of the tree follows that which is given in NCBI Taxonomy, so is not formulated based on the sequence alignments of hits from the protein queries. This differs from other tree tools and the reasoning behind this is that ProTaxoVis is primarily concerned with mapping presence onto the tree of life, a tree depicting evolution at the whole-organism level, therefore ProTaxoVis’s trees are not built based on queries and their hits are taken from a standard, in this case NCBI Taxonomy’s tree.

ProTaxoVis’s taxonomic tree branching covers all the organisms that contributed a hit to the protein query, with the number of total hits per protein represented as a colored slice of a pie chart. This pie chart is located at each node and scaled size-wise by the amount of hits mapping to the node. The tree is therefore a representation of the protein presence patterns mapped on a branching topology to show potential patterns in the protein’s evolutionary history, particularly in terms of gains or losses of the protein in various clades compared across the tree. ProTaxoVis’s *taxovis* tool creates one tree per protein, and a combined tree of up to three proteins, depending on the user’s choice. The upper limit of three is set such that the possible combinations of these proteins are representable in the pie chart (7 colors). *taxotree* is a wrapper tool that follows up *taxovis* to allow the user to prune and interact with the tree, and the configuration for trees is set in tree_config.txt and tree_to_prune.txt.

Whereas ProTaxoVis’s interactive heatmap allows for an overview of multiple proteins for a user-determined set of organisms, the taxonomic tree gives a comprehensive overview of presence-absence patterns as extracted from the sequence queries for a more focused set of proteins across the entire sampled tree of life. Therefore, the heatmap zooms in at the organism level and scales up at the protein level, while the taxonomic tree presents the exact opposite - large scale organism level representation on a zoomed in set of proteins. The questions that the tree answers are therefore more of the type related to evolutionary history of these proteins seen from a taxonomic branching perspective, and the tree thus complements the interactive heatmap.

## Results and discussion

To demonstrate a typical application of ProTaxoVis, we analysed the presence of six members of the phosphoribosyltransferase family. Phosphoribosyltransferases (PRTs) are important for the synthesis of nucleotides essential for DNA and RNA synthesis such as adenosine triphosphate ATP, uridine triphosphate UTP and metabolic cofactors such as NAD. We selected six of these enzymes: NAMPT and NAPRT (nicotinamide (Nam) and nicotinate (NA) phosphoribosyltransferase), QPRT (quinolinic acid (QA) phosphoribosyltransferase or nicotinate-nucleotide pyrophosphorylase), UPRT (uracil phosphoribosyltransferase), APT (adenine phosphoribosyltransferase), and HPRT (hypoxanthine-guanine phosphoribosyltransferase). NAMPT, NAPRT, and QPRT are involved in maintenance of NAD homeostasis and therefore crucial for cellular metabolism [30]. UPRT is involved in the pyrimidine salvage pathway, specifically by converting uracil into the pyrimidine nucleotide precursor uridine monophosphate [31]. Likewise, APT and HPRT are involved in the purine salvage pathway: APT converts adenine into adenosine monophosphate and HPRT converts hypoxanthine or guanine into inosine monophosphate or guanosine monophosphate [32, 33]. Phosphoribosyltransferase enzymes are essential to many organisms and therefore generally widely spread, with distinct distribution patterns across taxonomic groups that can be indicative of organism- or clade-specific pathway preferences, as is the case for the NAD enzymes and UPRT [21, 31]. This makes them an ideal test case for ProTaxoVis.

We seeded ProTaxoVis’s query step with a total of 23 sequences, purposely including well characterised and functionally confirmed proteins with, where applicable, at least one human and one bacterial sequence per enzyme, as seeding these two kingdoms samples two major groups of interest and allows for a more general comparison of distribution patterns. For several enzymes, plant and yeast seeds were available and used as well. All seed sequences, when possible, were taken from UniProt SwissProt, otherwise from TrEMBL. The seeds and their corresponding UniProt accession IDs are given in Table 1.

**Table 1:** **Seed Sequences:** Phosphoribosyltransferase seed sequences submitted as queries to *nr* protein database. The name, organism, and UniProt ID are given

Protein name	Source organism	UniProt ID
NAMPT	*Homo sapiens*	P43490
NAMPT	*Chlamydomonas reinhardtii*	D9I2J1
NAMPT	*Synechocystis sp.*	A0A068MXT3
NAPRT	*Homo sapiens*	Q6XQN6
NAPRT	*Drosophila melanogaster*	Q9VQX4
NAPRT	*Escherichia coli*	P18133
NAPRT	*Dictyostelium discoideum*	Q55G10
NAPRT	*Arabidopsis thaliana*	Q8RWM2
NAPRT	*Arabidopsis thaliana*	Q84WV8
QPRT	*Homo sapiens*	Q15274
QPRT	*Dictyostelium discoideum*	Q75JX0
QPRT	*Nicotiana tabacum*	A0A1S4CL59
UPRT	*Homo sapiens*	Q96BW1
UPRT	*Escherichia coli*	P0A8 F0
UPRT	*Saccharomyces cerevisiae*	P18562
APT	*Arabidopsis thaliana*	P31166
APT	*Homo sapiens*	P07741
APT	*Saccharomyces cerevisiae*	P49435
APT	*Escherichia coli*	P69503
APT	*Drosophila melanogaster*	P12426
HPRT	*Homo sapiens*	P00492
HPRT	*Dictyostelium discoideum*	Q54NJ8
HPRT	*Escherichia coli*	P0A9M2

Blast queries of our seed sequences against the May 2023 release of nr database returned a total of around 30,000 unique organisms (hit sequences), with no cross-hits between the protein sequences. If paralogues arising from recent gene duplication events are included in the analysis, one should adjust the e-value to a significance level lower than that at which cross-hits are identified. APT’s seed sequences retrieved the highest number of hits, approximately 14,000 hit sequences, followed by HPRT, UPRT, NAPRT, QPRT, and NAMPT, which had the fewest hits of around 3600 sequences. Whether these numbers are indicative of the abundance of these enzymes over all organisms, or simply a reflection of database scientific bias, is unknown. Our sampling, i.e. our choice of seed sequences and the number of seed sequences per enzyme, directly affects the number of hits. This effect is most likely not linear, as we see with our results and the mismatch between the ranking of the highest-hit-enzymes compared to how many seed sequences they had. For example, we were able to seed both APT and NAPRT comparably - with human, bacterial, and several other eukaryotic sequences. Both enzymes exhibit coverage in UniProt which is indicative of the broader scientific interest in these enzymes, yet NAPRT returned only half the hits in the larger nr database. This difference is interesting but not explainable without a comprehensive overview of enzyme coverage in current databases. What matters for the use of ProTaxoVis is the correctness of the seed sequences, and the user’s own prior biological knowledge, which determines the selection of organisms to contribute seeds.

### Distribution of NAD enzymes on the tree of life

**Fig. 3:**
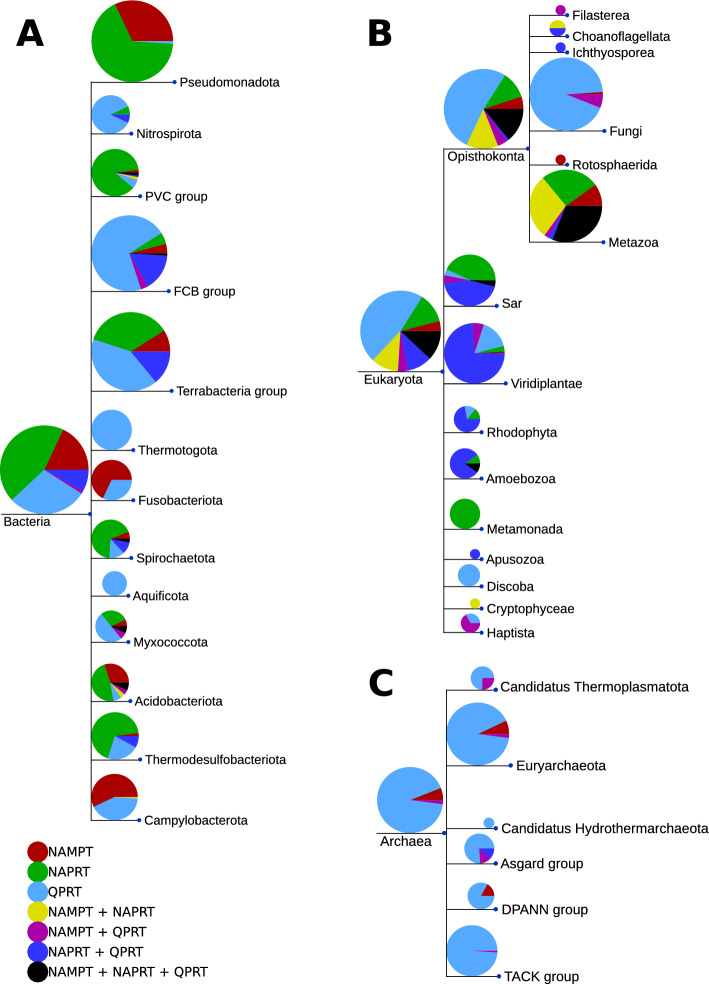
Taxonomic tree showing NAMPT, NAPRT, and QPRT distribution: Presence of NAMPT, NAPRT, and QPRT is mapped onto three NCBI taxonomic trees, bacterial, eukaryotic, and archaeal. The size of the pie charts is representing the number of organisms included. The charts show the relative number of hit sequences in each taxonomic group. Colors represent presence of a protein or combinations of these proteins indicated in the color legend at the bottom left. Bacteria (**A**) return the most hits and unique organisms, and show a mutually exclusive presence pattern for each of the three enzymes, or a preference for a combination of NAPRT and QPRT. Eukaryotes (**B**) exhibit a mixed distribution, with the Metazoa showing combinations of all three, NAMPT and NAPRT, or either one of these enzymes. Archaea (**C**) return the fewest hits and organisms, and of these organisms there is a strong presence of QPRT

The distribution of QPRT, NAPRT and NAMPT in bacteria appears to be in part mutually exclusive, potentially indicating repeated losses of the enzymes during bacterial evolution (see Fig. 3, panel A). Although these enzymes are all involved in NAD biosynthesis and show sequence similarities with each other, they can be clearly separated in our analyses with no cross hits. Major bacterial groups such as Terrabacteria, FCB, and PVC show a clear divide between clades returning hits for one of these NAD enzymes but not the others, and when multiple enzymes are found, it is usually NAPRT in combination with QPRT (shown as light blue pie chart wedges). With the exception of a small group in PVC and Acidobacteriota, NAPRT is not found co-existing with NAMPT in bacteria. It has been described earlier that although having similar sequences and structure, QPRT, NAPRT and NAMPT have particular differences in the catalytic core ensuring substrate specificity [34]. It has nevertheless been repeatedly shown that NAMPT can catalyze phosphoribosyl transfer to NA although with much lower affinity [35, 36]. This might explain the scattered distribution of these enzymes in bacteria although these enzymes are essential for their respective pathway, as these enzymes might be able to replace each other to a certain extent. Genome optimization in bacteria might have led to the selection of the enzyme for the most common substrate in the respective habitats, but alternative pathway functions have been maintained through substrate promiscuity.

The distribution of NAMPT, NAPRT, and QPRT in eukaryotes is much less clear-cut (see Fig. 3, panel B). Although the protozoan groups of Amoebozoa, Haptista, and Metamonada show preferences for single enzymes, too few sequence hits are returned overall for unicellular eukaryota to make a definite statement. For plants, on the other hand, NAPRT and QPRT are found in combination for the majority of organisms, with a few occurrences of NAMPT in combination with QPRT or just QPRT alone. The Opisthokonts show the most variety in the distribution of the three enzymes, but with a marked difference between fungal and metazoan organisms. Fungal organisms predominantly have QPRT, with some combinations of NAMPT and QPRT identified, whereas metazoan organisms have preferentially NAMPT or NAPRT, or a combination of both, or all three enzymes.

In Archaea (Fig. 3, panel C), which is arguably less well sampled than bacteria as measured by net sequence availability in the database, we find a dominance of QPRT-only organisms, with some NAMPT hits coming in between. This could also be a consequence of the lack of quality seed sequences for any of the PRTs from Archaea, which results in a lower coverage in this group.

ProTaxoVis’s taxonomic trees use these NAD enzymes to illustrate a multilevel, taxonomic representation of the presence-absence patterns, with the level tunable based on tree pruning and customisation. We show the combined tree, comparing all three enzymes, in Fig. 3, but there are individual trees per enzyme generated with the *taxotree* tool as well. The combined tree allows for an overview of enzyme evolutionary events, potential losses and gains, and is critical to guide further work towards this direction, both computationally and experimentally. This tree is best used to visualise combinations of up to 3 proteins, simply because the color-coding of protein abundance pie charts gets complex with more proteins, but nevertheless allows the user for full control to zoom in or out of taxonomic groups of interest.

### Distribution of PRTs on a heatmap

**Fig. 4:**
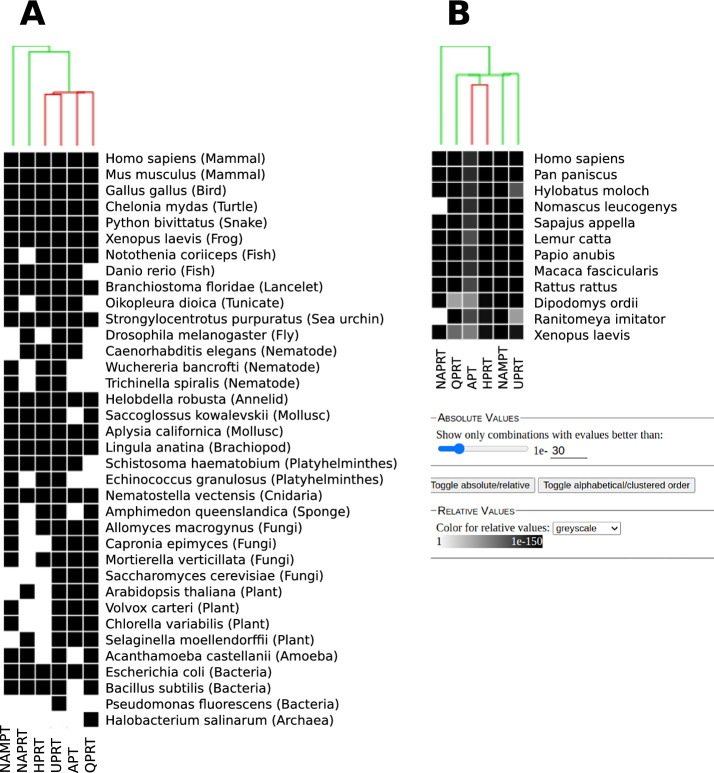
PRT Heatmap: Heatmap visualisation of presence patterns for the six PRT enzymes, with centroid hierarchical clustering of the hits shown. Organisms chosen are a set of common model organisms, mainly eukaryotic but with a few bacteria and one archaeal organism. Panel A shows a the absolute black-white view of the heatmap. The e-value cutoff for Panel A is left at default, 1e-30, which is taken as a not-too-stringent cutoff but still significant enough to return related hits. Panel B shows the relative heatmap view with a more closely related subset of vertebrates, with hit significance (E-value) to *Homo sapiens* protein seeds shown in grayscale. Sliders and toggle to switch between absolute and relative views and adjust e-value are shown in the bottom half of Panel B. Refer to description in General Workflow on heatmaps for detailed usage of heatmap views

To view the distribution of the additional PRTs: UPRT, APT, and HPRT across organisms, we use a heatmap representation (see Fig. 4). As a complement to ProTaxoVis’s taxonomic tree, the heatmap allows for comparison of presence patterns over many proteins, where the presence or absence of hits for each organism can be viewed in absolute (black or white) and toggled based on e-value significance of the hits (grayscale). Panel A in Fig. 4 shows strongly significant hit sequences for Metazoan organisms of all the PRTs, reflecting the Metazoan model organism seed sequences. This would represent a rough overview, where groups of homologous seeds to multiple proteins are used to probe if a subset of organisms have this protein, without regards to specifically which seed garnered a hit in which organism. All chosen organisms seem to have some form of UPRT present. NAMPT’s distribution pattern is more similar to NAPRT. The next step would be to subset the seeds by organism and regenerate the heatmaps to determine which seeds gathered which hits, and what this means about how related the protein from the seed organism is to putative homologs. Panel B in Fig. 4 then allows for a more targeted comparison view, where the human seed sequence is used to compare presence of PRTs in selected primates, rodents, and other model organisms, but only in the vertebrate clade. With this visualisation option that shows the e-value of the blast hit in grey scale. One can get an impression about the similarity or rate of change of each protein within a narrower evolutionary time frame.

The choice of organisms in this heatmap reflects an interest in the presence patterns of PRTs amongst select model organisms, mainly eukaryotic. In practice, while the user has full control of the organisms and taxonomic level on which to view presence patterns, the organisms are selected to span a certain clade of interest, or to specifically focus on a group of related organisms. Figure 4 thus gives an overview that is focused on spanning many proteins for a chosen set of organisms or taxa.

## Conclusion

ProTaxoVis is a novel bioinformatic tool that visualises protein presence patterns across select taxonomic groups or across all kingdoms. The tool collects, filters, and combines sequence query results for proteins of interest, and maps them onto the tree of life or as a heatmap of hit significances for a certain set of organisms. The tool was designed to provide a presence overview of enzymes and aid comparative pathway analysis, and has been used to study the NAD pathway [20, 21] as well as the mTOR pathway [22]. We show here that ProTaxoVis lends itself well to the analysis of several of the phosphoribosyltransferases (PRTs), as patterns can be seen in both the tree and heatmap visualisations. The taxonomic tree points towards bacteria harbouring NAD enzymes in a mutually exclusive manner, whereas eukaryotes have them in various combinations, which could strongly be linked to diversification of NAD recycling pathways in the latter group, as was mentioned in [20]. The heatmap compares six PRTs to show that distribution patterns of similar enzymes such as NAMPT and NAPRT over model organisms tend to match with comparable hit significance levels, while other PRTs have less of a distinct pattern.

## Availability and requirements

Project name: ProTaxoVis

Project home page: https://github.com/MolecularBioinformatics/ProTaxoVis

Operating systems(s): Platform independent

Programming language: Python

Other requirements: Python$-$3.5 or higher, wheel 0.33.0 or higher, numpy 1.15.1 or higher, scipy 1.11.0 or higher, matplotlib 3.1.1 or higher, pandas 1.0.0 or higher, Pillow 6.0.0 or higher, biopython 1.7.4 or higher, ete3 3.1.1 or higher, taxfinder 0.0.1.

License: MIT

Any restrictions to use by non-academics: None

## Data Availability

ProTaxoVis is an open-source software and available at: https://github.com/MolecularBioinformatics/ProTaxoVis. All files used in the PRT use case, including query results and configuration files, are made available in an additional repository, https://github.com/MolecularBioinformatics/ProTaxoVis-examples. We accessed the NCBI *nr* database in May 2023, corresponding to the April 2023 release. The current version of *nr*, which will include the April 2023 release, can be accessed at https://ftp.ncbi.nlm.nih.gov/blast/db/. At the time of writing, NCBI does not store archived versions of its blast databases on the FTP site. If the user wants to only query against the April 2023 version, they are directed towards the Entrez portal https://www.ncbi.nlm.nih.gov/search/ to subset the *nr* database with the April 2023 cutoff.

## Abbreviations

NAD: Nicotinamide adenine dinucleotide
NA: Nicotinic acid
mTOR: Mammalian target of rapamycin
NAPRT: Nicotinate phosphoribosyltransferase
PRT: Phosphoribosyltransferase
NAMPT: Nicotinamide phosphoribosyltransferase
QPRT: Quinolinate phosphoribosyltransferase
QA: Quinolinic acid
UPRT: Uracil phosphoribosyltransferase
APT: Adenine phosphoribosyltransferase
HPRT: Hypoxanthine phosphoribosyltransferase
FCB: Bacterial superphylum with Fibrobacteria, Chlorobiota, and Bacteroidota
PVC: Bacterial superphylum with Planctomycetota, Verrucomicrobiota, and Chlamydiota
